# CRABP1-complexes in exosome secretion

**DOI:** 10.1186/s12964-024-01749-w

**Published:** 2024-07-29

**Authors:** Jennifer Nhieu, Chin-Wen Wei, Megan Ludwig, Justin M. Drake, Li-Na Wei

**Affiliations:** Department of Pharmacology, University of Minnesota, 6-120 Jackson Hall, 321 Church St. SE, Minneapolis, MN 55455 USA

**Keywords:** CRABP1, Signalosome, Exosome secretion, Proteomics, Bioinformatics, Kinase, Actin

## Abstract

**Background:**

Cellular retinoic acid binding protein 1 (CRABP1) mediates rapid, non-canonical activity of retinoic acid (RA) by forming signalosomes via protein-protein interactions. Two signalosomes have been identified previously: CRABP1-MAPK and CRABP1-CaMKII. *Crabp1* knockout (CKO) mice exhibited altered exosome profiles, but the mechanism of CRABP1 action was unclear. This study aimed to screen for and identify novel CRABP1 signalosomes that could modulate exosome secretion by using a combinatorial approach involving biochemical, bioinformatic and molecular studies.

**Methods:**

Immunoprecipitation coupled with mass spectrometry (IP-MS) identified candidate CRABP1-interacting proteins which were subsequently analyzed using GO Term Enrichment, Functional Annotation Clustering; and Pathway Analysis. Gene expression analysis of CKO samples revealed altered expression of genes related to exosome biogenesis and secretion. The effect of CRABP1 on exosome secretion was then experimentally validated using CKO mice and a *Crabp1* knockdown P19 cell line.

**Results:**

IP-MS identified CRABP1-interacting targets. Bioinformatic analyses revealed significant association with actin cytoskeletal dynamics, kinases, and exosome secretion. The effect of CRABP1 on exosome secretion was experimentally validated by comparing circulating exosome numbers of CKO and wild type (WT) mice, and secreted exosomes from WT and siCRABP1-P19 cells. Pathway analysis identified kinase signaling and Arp2/3 complex as the major pathways where CRABP1-signalosomes modulate exosome secretion, which was validated in the P19 system.

**Conclusion:**

The combinatorial approach allowed efficient screening for and identification of novel CRABP1-signalosomes. The results uncovered a novel function of CRABP1 in modulating exosome secretion, and suggested that CRABP1 could play roles in modulating intercellular communication and signal propagation.

**Supplementary Information:**

The online version contains supplementary material available at 10.1186/s12964-024-01749-w.

## Introduction

Cellular Retinoic Acid Binding Protein 1 (CRABP1) is a highly conserved (> 99% amino acid sequence conservation) cytosolic protein previously suggested to play a role in binding retinoic acid (RA) for sequestration or channeling to cytochrome P450 family 26 (CYP26) proteins to regulate intracellular RA bioavailability [1]. RA, the principal active component of vitamin A, plays crucial roles in development, differentiation, and most physiological processes by binding to nuclear RA receptors (RARs), thereby regulating gene transcription. These effects involve alterations in gene expression, and therefore cannot be detected until hours or days later [2]. These activities are collectively referred to as “canonical” activities of RA. CRABP1 has been proposed to participate in this canonical RA signaling activity through binding RA to facilitate its catabolism.

However, experimental data have also shown certain rapid (within minutes) effects of RA, which occur mostly in a cell context-dependent and RAR-independent manner without altering gene expression. Recently, CRABP1 has been identified as the mediator of these rapid RAR-independent activities of RA detected in the cytosol, which involve specific context-dependent kinase signaling pathways. These are together referred to as “non-canonical” RA activities [3]. These genetic and molecular studies of CRABP1 have utilized, mainly, a *Crabp1* gene knockout (CKO) mouse model and primary tissues, as well as specific gain-of-function studies conducted using various cell culture systems. In brief, using CKO mice, as well as CKO embryonic stem cell (ESC) and primary tissues, these studies have revealed that CRABP1 can modulate ESC cell cycle progression [4], hippocampal neural stem cell (NSC) proliferation [5], cardiomyocyte’s sensitivity to isoproterenol assault [6], neuronal exosome release [7], adiponectin secretion from adipocytes [8], motor neuron differentiation [9] and the maintenance of neuromuscular junction (NMJ) [10], and the health of the thyroid gland [11]. In most of these studies where specific signaling pathways were determined, a common observation is the ability of CRABP1 to physically interact with and modulate specific kinase systems in a particular context. Importantly, human studies have also shown drastically altered expression of CRABP1 in various diseases such as neurodegeneration, autoimmune diseases, and cancers [12], supporting human disease relevance of the CKO phenotypes. The drastic dysregulation of *CRABP1* gene expression in human diseases clearly demonstrates the importance of CRABP1 in human health and diseases.

As introduced earlier, CKO mouse studies have revealed a common phenomenon that CRABP1’s function is related to specific signaling complexes, particularly kinases, mostly through its direct physical association with specific components in the signaling cascade. As such, we have proposed that CRABP1 forms various, specific “signalosome complexes” in cells expressing CRABP1 in order to timely modulate specific signaling pathways. Through extensive studies we have previously established two CRABP1-signalosomes. The first consists of CRABP1 and its direct interaction partner, Raf-1 kinase; formation of CRABP1-Raf-1 signalosome dampens Ras-triggered mitogen-activated protein kinase (MAPK) pathway activation [13], and ultimately modulates various normal cellular processes and cancer cell apoptosis [14]. The second consists of CRABP1 and its direct interaction partner, Ca^2+^/calmodulin-dependent protein kinase II (CaMKII); formation of CRABP1-CaMKII signalosome dampens calmodulin (CaM)-induced CaMKII enzyme activation [15], and ultimately protects cells from toxicity induced by over-activated CaMKII [6]. Given the wide disease spectrum of CKO mice, it is highly possible that additional CRABP1 signalosomes exist. To uncover additional novel CRABP1-signalosomes operating in various cells/tissues by using conventional experimental systems can be very laborious. A more efficient and comprehensive approach is highly desirable.

The “CRABP1 signalosome” theory prompted this current study. We exploited a combinatorial approach, starting with a mass spectrometry (MS)-based screening to identify all the possible binding partners of CRABP1, which was followed by bioinformatic analyses to uncover potential pathways or cellular processes involved, and, finally, results were validated using experimental data collected from specific and defined experimental context/conditions. Through these series of systemic investigations, this current study provides the first proof-of-concept for the power of this novel combinatorial strategy. The data presented here have uncovered additional novel mechanistic details, signaling pathways, and physiological processes involving CRABP1, such as in regulating exosome secretion.

## Results

### Proteomic analysis of CRABP1-Interacting protein complexes

Previously we have conducted in-depth molecular and biophysical studies to characterize the structural basis underlying the formation of CRABP1-MAPK and CRABP1-CaMKII signalosomes [13, 15]. As introduced, this current study aimed to more comprehensively identify novel CRABP1-interacting proteins on a larger scale in order to more efficiently and comprehensively uncover novel biological functions and mechanisms of action of CRABP1. We first employed immunoprecipitation combined with mass spectrometry approaches (IP-MS) to screening for and identify CRABP1-interacting proteins in a CRABP1-expressing HEK293T cellular background because of its high transfection efficiency [16]. This cell line, derived from the HEK293 cell line [17], has been widely used in proteomic studies, providing a dearth of publicly available data important for candidate and false positive screening analyses (see later).

**Fig. 1:**
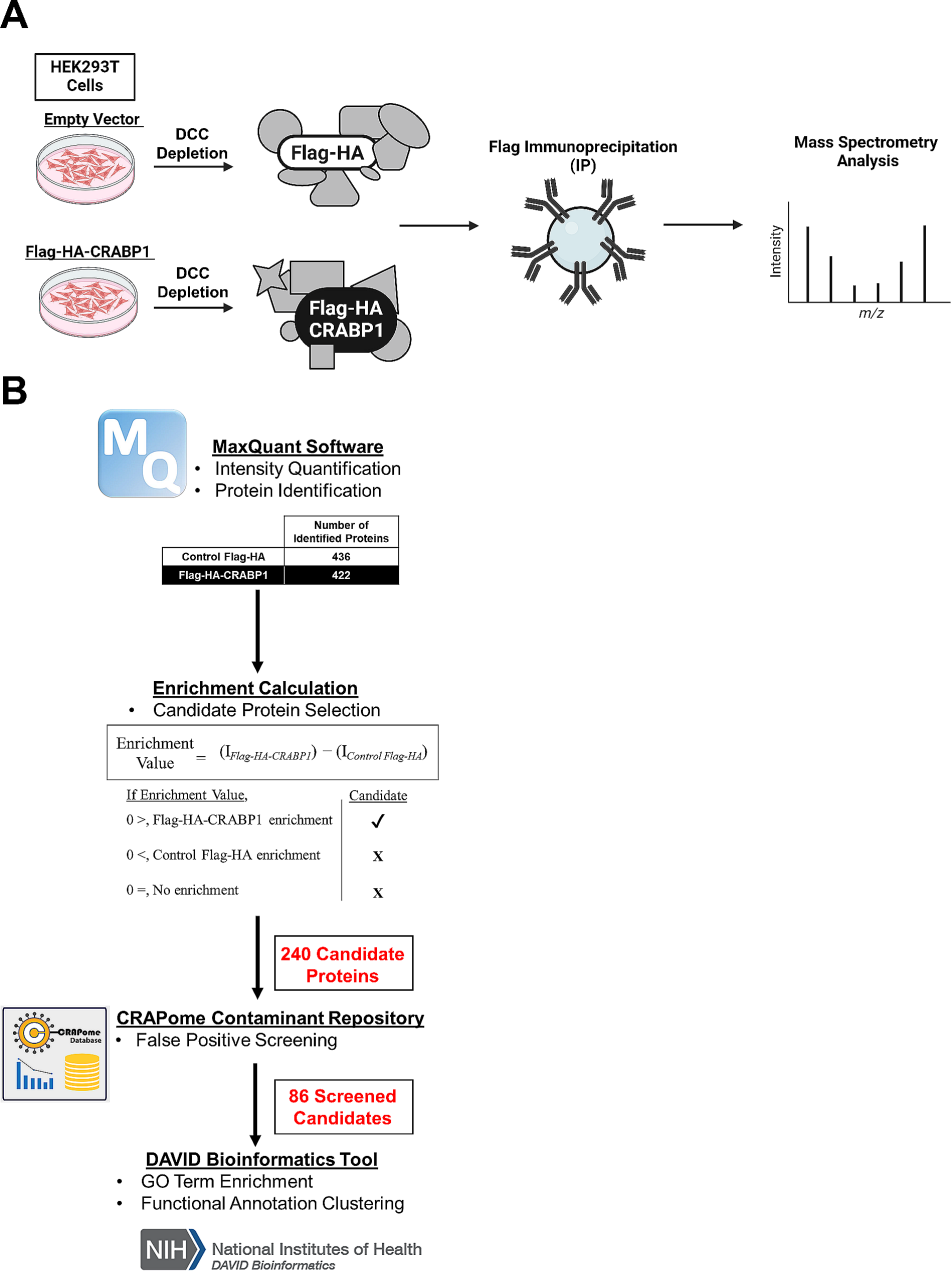
IP-Mass Spectrometry (IP-MS) experimental scheme and bioinformatic analyses. **(A)** For immunoprecipitation (IP)-mass spectrometry experiments, HEK293T cells were transfected with Flag-HA-tagged CRABP1, or with an empty vector expressing only Flag-HA as a negative control (Control Flag-HA, CF). DCC depletion to remove endogenous hormones and lipids, including atRA was performed 24 h prior to Flag-IP. Flag-IP was then performed using Flag-HA-CRABP1 and Control Flag-HA cell lysate. IP samples were then processed and subjected to MS analysis. **(B)** Raw MS data was processed by MaxQuant to identify proteins and signal intensities. For candidate protein selection, enrichment values were calculated from normalized intensity differences between Flag-HA-CRABP1 intensity (I_Flag−HA−CRABP1_) minus Control Flag-HA intensity (I _Control Flag−HA_). Candidate proteins were defined as having an Enrichment Value > 0. A total of 240 candidate proteins were identified. Candidate proteins were then screened for false positives using the CRAPome Contaminant Repository. A total of 86 screened candidates were then bioinformatically analyzed for GO term enrichment and functional annotation clustering using the DAVID Bioinformatics tool

HEK293T cells were first transfected with Flag-HA-tagged CRABP1 (Flag-CRABP1), or with an empty vector expressing Flag-HA as a negative control (CF). In order to circumvent confounding factors from endogenous atRA, transfected HEK293T cells were exchanged into dextran-charcoal-treated (DCC) median to deplete endogenous RA. Cells lysates were then subjected to Flag-IP to capture protein complexes. Flag-IP captured protein complexes were then subjected to MS proteomic analysis (Fig. 1A).

Following analyses of MS data that revealed CRABP1-interaction proteins, targets were further analyzed using bioinformatic tools to gain insights into their biological functions, presumably involving CRABP1-containing complexes (Fig. 1B). First, the raw MS-IP proteomic data, was processed by MaxQuant software [18] for peptide searching, protein identification, and intensity quantification. Using 1% false-detection rate (FDR), 436 proteins were identified in the CF condition and 422 proteins were identified in the Flag-CRABP1 condition. Intensity quantification followed by normalization and imputation allowed for enrichment value calculations to identify candidate proteins that formed complexes with CRABP1. CRABP1-interaction candidate status was calculated by taking the numerical difference between the protein intensity values of Flag-CRABP1 (I_Flag−CRABP1_) minus CF (_Control Flag−HA_). An enrichment value > 0 defined a protein as a CRABP1-interaction candidate. A total of 240 candidate proteins were identified as potential CRABP1-interaction partners. In order to rule out potential false positives, we further screened these 240 candidates using the CRAPome Contaminant Repository. The CRAPome Repository contains a substantial collection of negative control IP-MS experiments, allowing users to identify proteins for their spectral abundance in negative control experiments according to the desired experimental condition [19]. A false positive was defined as having a spectral count within the top 4th quartile of at least three independent experiments and subsequently removed. After false-positive screening, a total of 86 screened candidate remained for further bioinformatic characterization. A complete protein list from IP-MS experiments can be found in Additional File 1: Supplementary Table 1.

### Bioinformatic characterization of CRABP1-interaction complexes

These 86 candidate proteins were then subsequently analyzed using bioinformatic tools to gain insights into their potential functions. First, CRABP1-candidate proteins were submitted to the Database for Annotation, Visualization and Integrated Discovery (DAVID) web server [20, 21] for GO Term Enrichment analysis [22, 23] to identify and rank enriched terms within the Biological Process (BP), Cellular Component (CC), and Molecular Function (MF) GO domains. These GO domains represent the broad classifications associated with each individual GO term [22]. To reduce redundancy and to gain biological insights, terms from each GO domain were organized into ranked, functional clusters using Functional Annotation Clustering analysis [21]. Ranked clusters were scored using an “Enrichment Score” from which biological relevance could be inferred according to the magnitude of the score [21]. Additionally, within each cluster, the individual GO terms were ranked by significance.

**Fig. 2:**
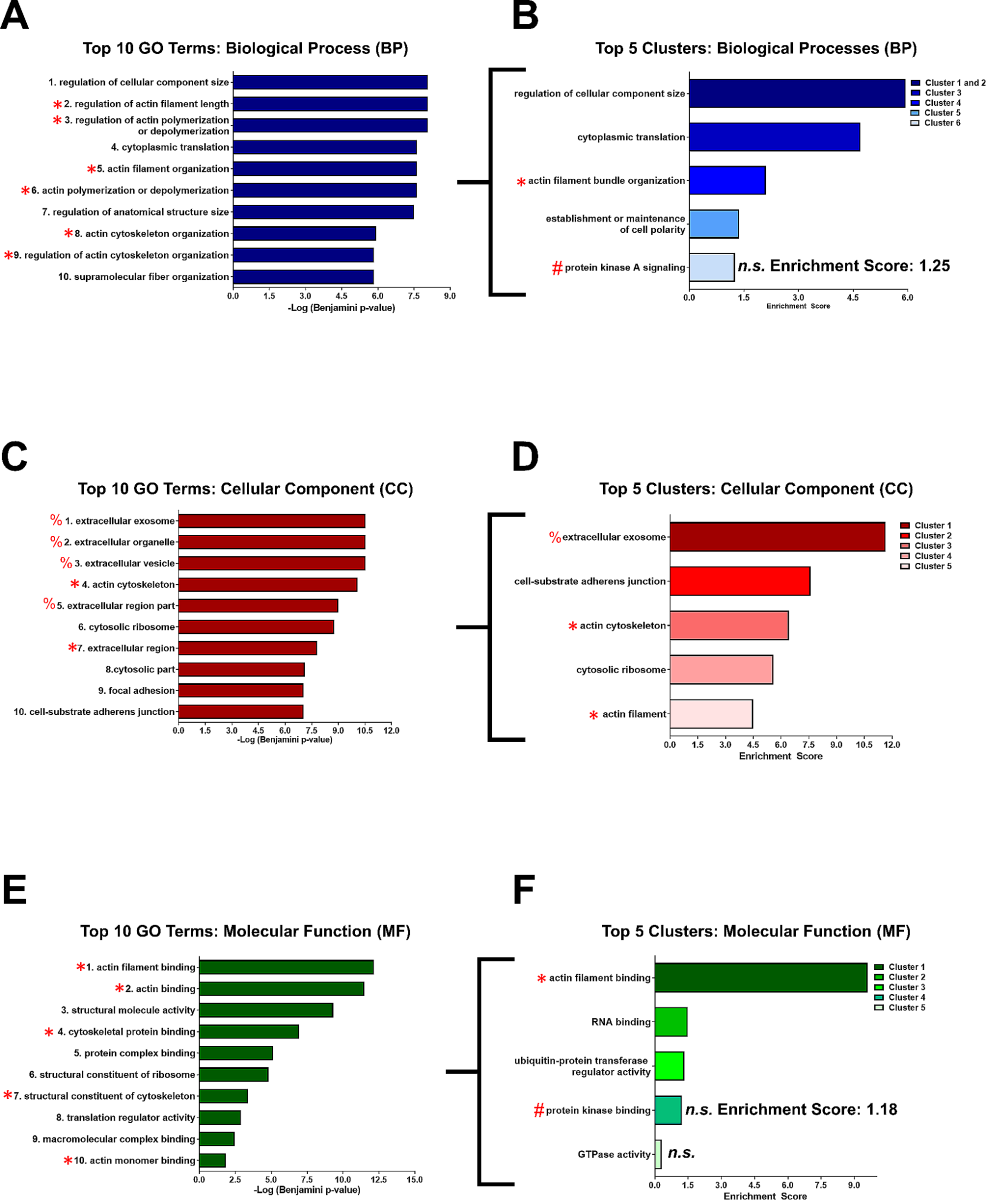
GO Term Enrichment and Functional Annotation Clustering of candidate proteins. **A-C)** Left plots: Top 10 enriched GO terms identified by GO term enrichment analysis for Biological Process (BP) **(A)**, Cellular Component (CC) **(C)**, and Molecular Function (MF) **(E)**. Right plots: Top 5 GO Clusters identified with functional annotation clustering for BP **(B)**, CC **(D)**, and MF **(F)** terms. Brackets indicate that the GO terms used in Functional Annotation Clustering are derived from the initial GO term enrichment analysis. Red asterisks mark terms related to actin dynamics. Red pound signs mark terms related to kinases. Red percent signs mark terms related to extracellular and exosome components. Unless marked by “n.s”, all terms and clusters ranked as significant. Significance for GO terms is defined as a Benjamini corrected *p*-value of ≤ 0.0, and an Enrichment Score of 1.3 is used for functional clusters

GO term enrichment identified the following number of significant (*p* ≤ 0.05) GO terms for each domain: 72 BP terms, 47 CC terms, and 14 MF terms. Upon Functional Annotation Clustering, the following number of significant (enrichment score ≤ 1.3) clusters were identified for each domain: 5 BP clusters, 10 CC clusters, and 3 MF clusters. A complete list of GO term enrichment and functional annotation clustering results can be found in Additional File 2: Supplementary Table 2. The Top 10 GO terms (left plots) and Top 5 functional clusters (right plots) from each BP, CC and MF GO domain were presented in Fig. 2. Functional clusters were named according to the top-ranked term within each corresponding cluster. The bracket ([) denotes that the functional clusters are derived from the enriched GO terms.

Under the BP domain, a majority of the Top 10 enriched terms were related to actin dynamics (Fig. 2A, left plot, Terms 2, 3, 5, 6, 8, 9). Additionally, the top ranked term, “regulation of cellular component size” (Term 1, GO:0032535), GO is a parent term to “regulation of actin filament length” (GO:0030832). Therefore, actin is also a relevant aspect of this number 1 ranked term (Term 1). Upon functional clustering, actin regulation was also apparent as a top-ranked functional cluster (Fig. 2B, right plot, Cluster 3). Clusters 1 and 2 exhibited a high degree of overlap in GO terms and were therefore represented by a single bar in Fig. 2B. Interestingly, a cluster related to kinase signaling also ranked highly (Cluster 6, Enrichment Score; 1.25) with an enrichment score approaching the significance cut-off of 1.3. Under the CC domain, the Top 10 enriched terms were related to extracellular vesicles and the extracellular space (Fig. 2C, Terms 1, 2, 3, 5, 7) and actin (Fig. 2C, Term 4). Extracellular exosomes ranked as the top cluster (Fig. 2D, Cluster 1) and actin filament ranked as a top 5 cluster (Fig. 2D, Cluster 5). Under the MF domain, several Top 10 terms were related to actin binding (Fig. 2E, Terms 1, 2, 4, 7,10). Upon functional clustering, actin filament binding (Fig. 2F, Cluster 1) ranked as the top cluster. Additionally, protein kinase binding (Fig. 2F, Cluster 4) also emerged as a top-ranked cluster (enrichment score 1.18). Complete GO Term Enrichment and Functional Annotation Clustering results are available in Additional File 2: Supplemental Table 2.

In summary, GO term enrichment followed by functional annotation clustering revealed regulation of actin dynamics as a major theme associated with these 86 CRABP1-interacting proteins (Fig. 2A-F, red asterisks). The CC enriched terms also suggested a strong association with the extracellular compartment, in particular exosomes (Fig, 2 C-D, red percent signs). This is consistent with our previous experimental results revealing altered circulating exosome profiles in CKO mice [7]. Additionally, the appearance of kinase related terms (Fig. 2B and E, red pound signs) also supports the known CRABP1-kinase relationship that has been experimentally revealed, such as CRABP1-MAPK [13] and CRABP1-CaMKII [15] signalosomes. Other significant enriched terms and clusters thematically present amongst the BP, CC, and/or MF domains include terms related to cytosolic ribosomes and translation (Fig. 2-D) and ubiquitin activity (Fig. 2F). The implications of these other terms are further discussed later (Discussion).

These results support the notion that there are additional novel functional roles for CRABP1 in forming signaling complexes which can modulate various biological processes. One such process, as revealed from the above analyses, is the regulation of exosome profiles by CRABP1-signalosomes that could modulate actin dynamics and/or the activation of specific kinase cascades.

### Deletion of Crabp1 impairs exosome biogenesis and secretion

**Fig. 3:**
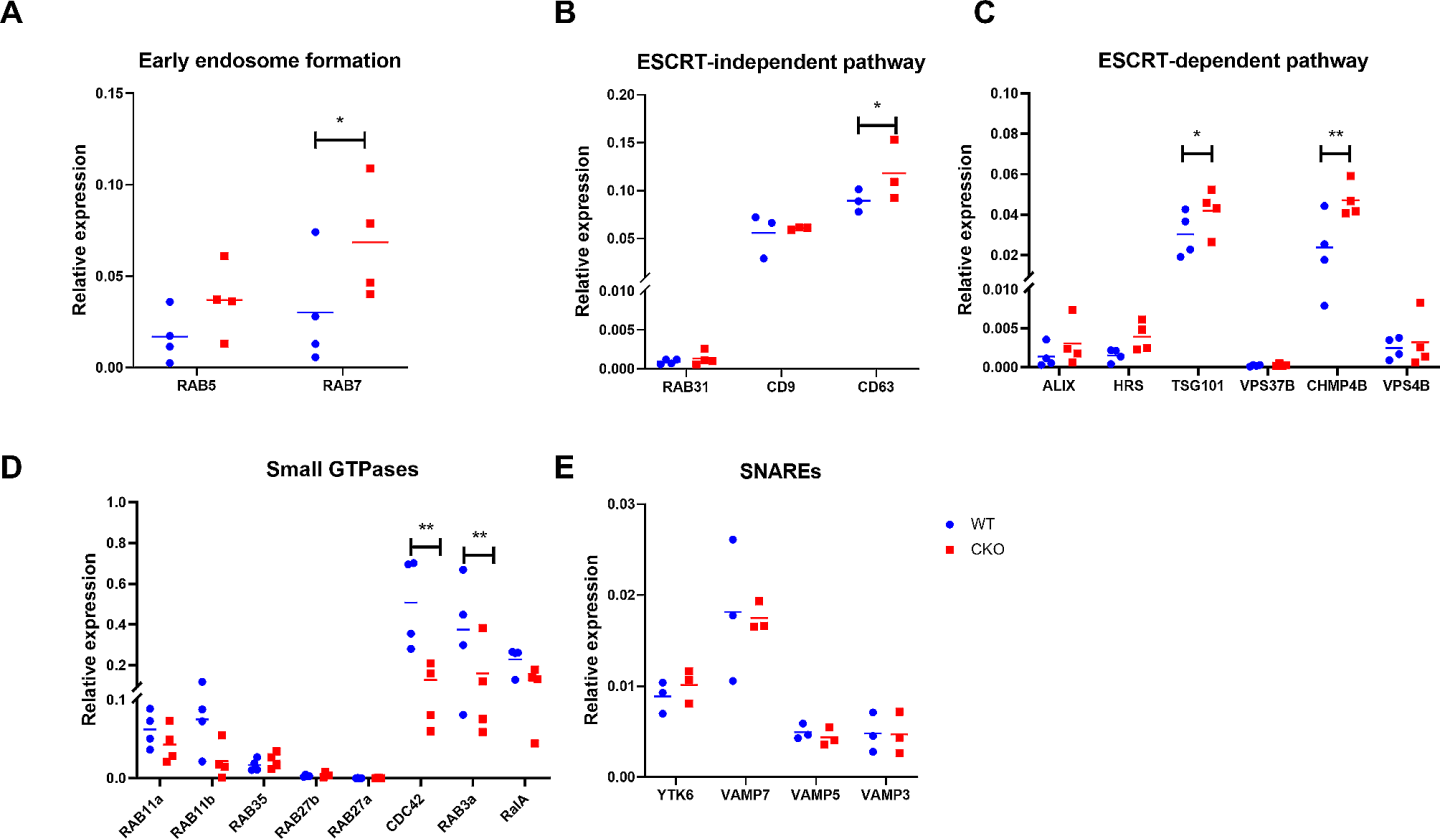
Gene expression profiling of wild-type (WT) and CRABP1-knockout (CKO) spinal cord tissues. qPCR to determine the expression of genes related to early endosome formation **(A)**, ESCRT-independent pathway **(B)**, ESCRT-dependent pathways **(C)**, small GTPases **(D)**, and SNAREs **(E)** in spinal cord tissues. Normalization to RPL19 was used an internal control. Student’s t-test, *p < 0.05

The above analyses revealed a potential role for CRABP1 in the process of exosome biogenesis or secretion. Interestingly, the CKO mice indeed showed an altered circulatory exosome profile as compared to wild type mice [7]. We thus designed experiments to validate the causal relationship of CRABP1 and exosome secretion. We first exploited the readily available CKO mice, and examined the expression of genes associated with exosome formation in their spinal cord tissues where MNs (which express CRABP1 highly and can secret neuronal exosomes) reside. As shown in Fig. 3A, there was a moderate increase in the expression of *RAB5* and a significant increase in *RAB7*, both are crucial to early endosome formation [24], in CKO’s spinal cord tissue as compared to WT tissue. Subsequently, we analyzed the expression of genes related to both ESCRT-independent (Fig. 3B) and ESCRT-dependent (Fig. 3C) pathways [25, 26]. Clearly, between CKO and WT spinal cord tissues, there was a significant increase in CD63 expression in CKO. Moreover, in CKO spinal cord tissue, the expression of genes involved in the ESCRT-dependent pathway, such as *ALIX*,* HRS*,* TSG101*, and *CHMP4B*, was increased. In particular, *TSG101 and CHMP4B* exhibited most significant upregulation among these genes.

We next compared the expression of genes associated with exosome secretion, which predominantly involves SNAREs and small GTPases [25, 26]. The data showed no difference in SNAREs gene expression in the spinal cord between WT and CKO mice (Fig. 3E). However, in CKO tissue, there was a trend towards reduced expression of *RAB3a* and *RAB11*, both are pivotal Rab GTPases involved in regulating fusion or docking of multivesicular bodies (MVBs) with the plasma membrane [27, 28]. Moreover, there was a significant reduction in the expression of exocytosis-related genes, including *CDC42* and *RalA* [29] in CKO (Fig. 3D). Taken together, changes in the expression of these genes in CKO further support that Crabp1 deficiency impairs the biogenesis and/or secretion of exosomes, as reflected in the consistently altered expression of genes related to exosome biogenesis/secretion. The altered gene expression patterns in CKO tissues confirm a shift in the physiological context of CKO where exosome synthesis/secretion pathways indeed are disrupted.

**Fig. 4:**
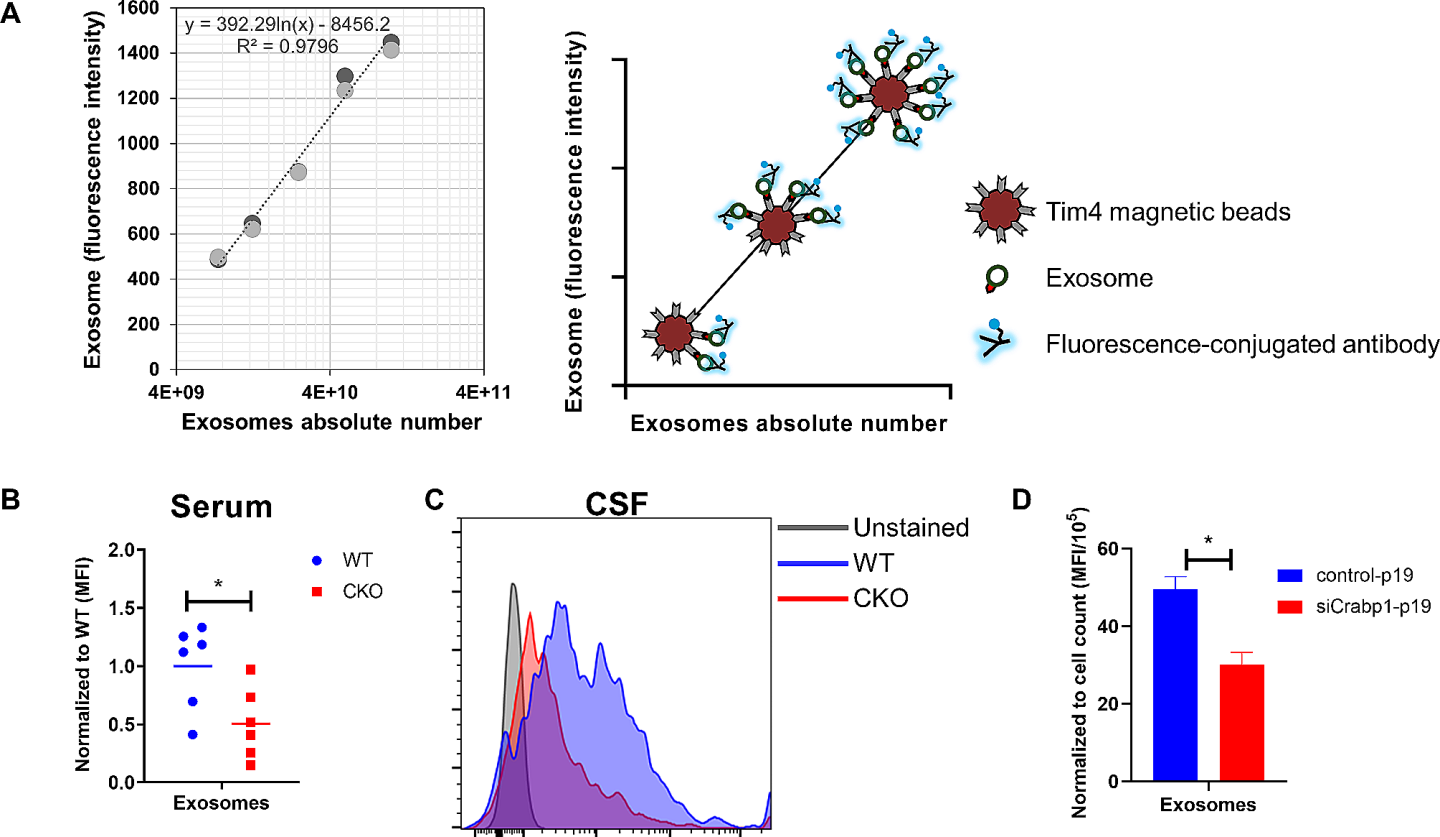
Functional studies of CRABP1 in exosome secretion. The function of CRABP1 in exosome secretion was determined by comparing exosome numbers in mouse (WT vs. CKO) serum and CSF, as well as exosome numbers secreted from P19 control and a P19-CRABP1 knock-down cell line. **(A)** Illustration of exosome quantification by bead-based flow cytometry approach (Right panel). Standard Curve for exosome qualification shows that increasing exosome concentration results in increased fluorescence intensity (Left panel). **(B)** Flow cytometry analyses of exosomes collected from WT and CKO mouse sera. CKO data were normalized to WT, and the pooled results were from two independent experiments. **(C)** Representative flow cytometry histograms of exosomes collected from mouse WT and CKO CSF samples. Pooled results were from two independent experiments. **(D)** Flow cytometry analyses of exosomes collected from culture supernatant of P19 Control and siCRABP1-P19 cells. Error bars show means ± SD. Student’s t-test, **p < 0.01. MFI = Mean fluorescence intensity

We next quantitatively validated that CRABP1 deficiency has impaired secretion of exosomes. In this experiment, we quantified exosome numbers in sera from WT and CKO mice using a bead-based flow cytometry. We utilized Tim4-labeled beads (FUJIFILM Wako Chemicals), which selectively bind to phosphatidylserine on exosome surfaces, then labeled exosomes with anti-CD9 fluorescence. To validate this method, we first determined the absolute exosome counts using Nanoparticle tracking analysis (NTA). Figure 4A illustrates that as exosome concentration increases, more fluorescently labeled exosomes bind to beads, resulting in increased fluorescence intensity.

Figure 4B shows the data of the mean fluorescence intensity (MFI). It appears that exosome number was indeed lower in CKO serum, confirming a reduction in the number of exosomes secreted by CKO tissues. Consistently, the quantified result of exosomes in the cerebrospinal fluid (CSF) of CKO mice (Fig. 4C) was also lower in CKO group, demonstrating a consistently reduced number of circulating exosomes in CKO mice. The fact that gene expression of exosome secretion in CKO was suppressed (Fig. 3D) would also support that depleting CRABP1 impaired exosome secretion. To further validate if changes in exosome numbers in CKO indeed was the result of malfunction in cells that secrete exosomes, i.e. a cell-autonomous event, we established a CRABP1-knockdown P19 cell line (siCRABP1-P19) which otherwise would express abundant CRABP1 endogenously. This experiment aimed to demonstrate whether *Crabp1* deficiency in P19 cells could affect the number of exosomes secreted from these cells. As shown in Fig. 4D, siCRABP1-P19 cells secreted much fewer exosomes.

These findings validate that *Crabp1* deficiency indeed reduced the number of secreted exosomes, consistent with the reduced numbers of circulating exosomes in the biological fluids of CKO mice. While our results have shown that Crabp1 deficiency seemed to also impair biogenesis of exosomes, as evidenced by the upregulation of genes associated with exosome formation in CKO mice when compared to WT, the complexity of exosome biogenesis and its numerous regulatory pathways make it difficult to conclude to what extent CRABP1 impacts this particular process. However, the consistently decreased expression of genes related to exosome secretion pathways in CKO mice, as compared to WT, would support that Crabp1 plays a pivotal role in modulating the release of exosomes. These experimental results confirm the above bioinformatic analyses, and conclude that CRABP1 can function to modulate exosome secretion (Fig. 2D).

### Pathways for CRABP1 in exosome biogenesis and secretion

These series of experiments, as described above, MS-IP screening, bioinformatic analyses and experimental validation have identified novel CRABP1-signalosomes that can function in exosome biogenesis and/or secretion. To identify potential mechanisms/pathways for the function of CRABP1-signalosome complexes in exosome biogenesis and/or secretion, we performed pathway analysis using Kyoto Encyclopedia of Genes and Genomes (KEGG) [30–32] and Biocarta [33] databases. Enriched pathways known to be associated with exosome secretion provide evidence towards potential mechanisms/pathways where CRABP1-complexes may function. The KEGG pathway analysis identified two pathways known to be involved in exosome secretion, actin cytoskeleton and endocytosis. Biocarta pathway analysis identified PI3K subunit p85 in the regulation of Actin Organization and Cell migration, Y branching of actin filaments, and Rho cell motility signaling pathway. Upon inspecting the CRABP1-interacting protein candidates that could be associated with these pathways, ACTR3, ACTR2, ARPC3, and ARPC4 proteins appeared in all these pathways, comprising 4 out of the 7 members of the Arp2/3 protein complex. Arp2/3 is a highly conserved protein complex that functions to regulate various aspects of actin cytoskeleton by inducing the nucleation of actin into Y-branched networks, which is especially important in endosome maturation [34]. Most importantly, the Arp2/3 complex has been directly implicated in exosome secretion through binding and regulating cortactin [35]. Upon Arp2/3 binding, cortactin acts to stabilize the docking of multivesicular endosomes containing exosomes destined for secretion to the plasma membrane. Furthermore, as shown in Fig. 4C, CDC42 gene expression was found to be disturbed in CKO spinal cord tissue. CDC42 protein is an upstream regulator of Arp2/3, which ultimately affects actin cytoskeleton dynamics and endosome maturation during endocytosis [36].

**Fig. 5:**
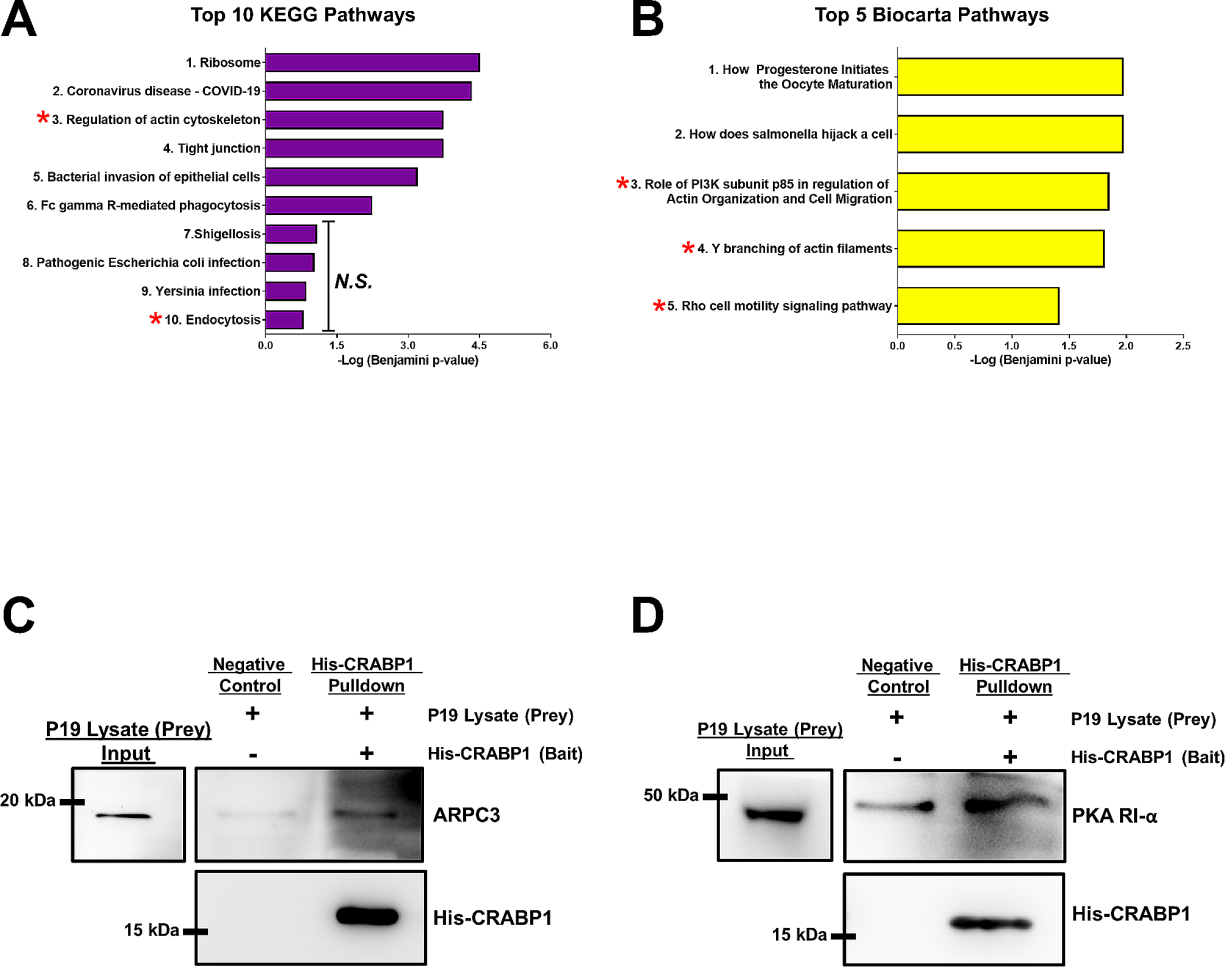
Pathway analysis and validation of CRABP1-protein complex formation in exosome secretion. A-B) KEGG pathways analysis **(A)** and Biocarta pathway analysis **(B)** of CRABP1-enriched protein identified by IP-MS. “n.s.”, not significant. Unless indicated by n.s. all pathways scored as significant, which was defined as a Benjamini corrected *p*-value of ≤ 0.05. Red asterisks mark exosome related pathways. **C-D)** His-CRABP1 Pull-down assay for ARPC3 **(C)** and the regulatory subunit of PKA (PKA RI-alpha) **(D)**. Top left: Input western blot of P19 cell lysate indicating the expected molecular weight for ARPC3 **(C)** and PKA RI-alpha, **(D)** Top right: Western blot for His-CRABP1 pulldown assay of ARPC3 **(C)** and PKA RI-alpha **(D)**. Bottom right: Levels of His-CRABP1 present in each pulldown reaction. Reactions containing only prey proteins served as the negative control. ARPC3 was detected by anti-ARPC3 antibody. PKA-RI alpha was detected by anti-PKA RI-alpha antibody. His-CRABP1 was detected by anti-His antibody

We previously have observed that CRABP1-modulated MAPK activity could affect the secretion of RIP140-containing (pro-inflammatory) exosomes in CKO mice [7]. Other studies have also indicated MAPK signaling in exosome biogenesis and secretion [37]. The current bioinformatic findings also show that kinase signaling and binding are enriched amongst the CRABP1-interacting proteins. All of these results support that CRABP1, through direct interactions with certain kinases, plays functional roles in exosome biogenesis and/or secretion. Interestingly, a PI3K pathway was identified during Biocarta pathway analysis (Fig. 5B, Pathway 3), and there existed functional clusters associated with kinase signaling and binding, From the CRABP1-interacting protein list in the functional clusters, we also identified kinases (PRKACA), kinase scaffold proteins (GLRX3, SFN, RACK1, TWF2) kinase modulators (PPP1CB, PIN1), and kinase substrate proteins (EZR, RDX, MSN, HNRNPA0). Several of these kinase and kinase-associated proteins have been determined to play a direct or indirect role in exosome biogenesis and secretion, as shown in Table 1, including PKA, PKC and PI3K, and MAPK, all have been shown to be involved in exosome secretion [38–40]. PKA was found to mediate the release of TNFR1, exosome-like vesicles from HUVEC cells. PKC activation regulates exosome secretion from T lymphocytes [40]. Loss of PI3K results in a decrease in levels of WNT10b-containing exosomes [39]. These pathway analyses show that CRABP1 can act to modulate exosome secretion by forming signalosome complexes to regulate specific kinase activities, such as PKA, PKC, or PI3K. Additionally or alternatively, it may act indirectly through modulating kinase scaffold or modulatory proteins.

As mentioned, pathway analysis of IP-MS results has identified components of the Arp 2/3 complex and kinases/kinase-related proteins as CRABP1-interactions that potentially function to regulate exosome secretion. These include a component of PKA, PKA-RI alpha, and Arp2/3 (Additional File 1, Supplementary Table 1). Both Arp 2/3 and PKA function in regulating exosome secretion [35, 38]. We thus examined these CRABP1-complexes in a biological background relevant to exosome secretion such as the P19 system (shown earlier in Fig. 4D). As shown in Fig. 5, CRABP1-ARPC3 (Fig. 5C) and CRABP1-PKA R1-alpha (Fig. 5D) complexes were detected in the P19 system. Note that background signal in negative controls is a known phenomenon in His-Tag pull-down assay (see Methods). These data validate that CRABP1 forms protein complexes with Arp 2/3 and PKA components in a biological system where CRABP1 plays a role in the regulation of exosome secretion.

**Fig. 6:**
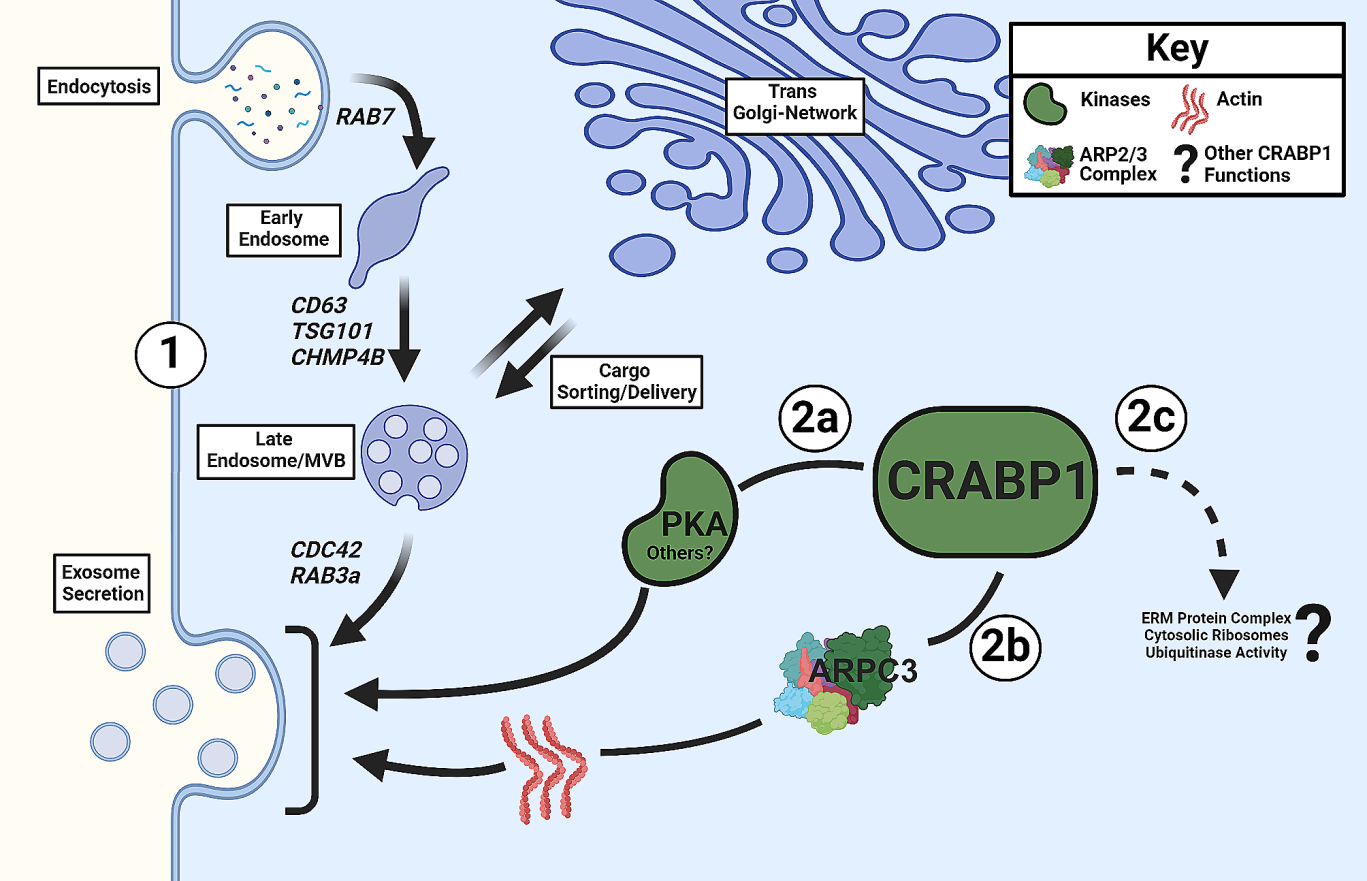
Proposed role of CRABP1 in exosome secretion. Circle 1: CRABP1 participates in the regulation of exosome biogenesis/secretion. Deletion of *Crabp1* disrupted exosome secretion, resulting in a physiological context consistent with aberrant expression of genes related to exosome biogenesis and secretion. The major processes involved in exosome biogenesis are represented by faded arrows. Specifically, genes associated with early endosome formation (RAB7), late endosome/multivesicular bodies (CD63, TSG101, CHMP4B), and exosome release (CDC42, RAB3a) were affected. Circles 2a-c: Proposed CRABP1-protein complexes that modulate exosome secretion. This study validated CRABP1 complex formation with PKA (Circle 2a); and a component of the ARP 2/3 complex, ARPC3 (Circle 2b) (solid arrows). Other kinases may also complex with CRABP1 to ultimately regulate exosome secretion (see Discussion). Additional CRABP1-protein complexes identified from IP-MS data include the ERM protein complex, cytosolic ribosomes, and ubiquitinase activity. These can also potentially be involved in exosome release. However, these remain to be examined (Circle 2c, dashed arrow with a question mark). This figure was created using Biorender.com

Figure 6 provides a summary of the new finding that *Crabp1* plays a role in exosome biogenesis and/or secretion, reflected by the dysregulated exosome secretion and disturbed expression of genes related to exosome biogenesis/secretion (Circle 1). At least two CRABP1-complexes were validated, providing mechanisms by which CRABP1 may participate in the regulation of exosome secretion: PKA (Circle 2a, solid arrow) and the ARPC3 of the Arp2/3 complex (Circle 2b, solid arrow). There were additional enriched terms related to translation and ribosomes, ubiquitination, and members of the ERM complex (Circle 2c, dashed arrow to indicate their speculative nature). Both ribosomes and ubiquitination are proposed to be important for exosome cargo composition and sorting [41, 42]. ERM proteins are also actin-binding proteins that affect the shape of plasma membrane and exosome biogenesis and secretion [43]. These additional terms and protein complexes provide insights for further studies about additional mechanisms through which CRABP1 may function.

## Discussion

We first identified a collection of candidate proteins that could form immunocomplexes with CRABP1 by using IP-MS. Bioinformatic analyses of these candidate proteins utilizing Gene Ontology (GO) enrichment and functional clustering were then applied. Based on GO term enrichment and functional clustering, the data revealed that these potential signalosomes were significantly associated with biological and molecular processes related to pathways of actin cytoskeleton, exosomes and extracellular compartments. We then validated these first findings with a particular emphasis on exosome secretion, because CKO mice did exhibit an abnormally secreted exosome profile (7). We thus conducted gene expression analyses of CKO and wild type (WT) mouse spinal cord tissues where MNs, CRABP1-expressing cells as the major neuronal exosome source, resided. We then further quantitatively compared exosomes in CKO and WT sera and CSF. Finally, we compared wild type and *Crabp1*-defeicient mouse cell line P19. The results showed that *Crabp1* deficiency indeed impaired the secretion of exosomes in both primary cells/tissues and cultured cell lines. These findings collectively validated that CRABP1 indeed plays a crucial role in regulating exosome secretion.

The IP-MS experiments were performed without atRA. Thus, formation of these complexes should be considered in the context of ligand-free CRABP1 (apo-CRABP1). In future experiments, it would be of great interest to study how atRA may further alter the function of CRABP1 signalosomes. These CRABP1-signalosomes are expected to be highly dynamic in terms of their composition and/or assembly in various physiological contexts. This is especially evident as shown in our earlier studies where CRABP1 were found to form various signalosomes in different cell types, such as CRABP1-MAPK signalosome in stem cells versus CRABP1-CaMKII signalosome in excitable cells like cardiomyocytes and motor neurons [3, 12]. It would be interesting to apply the IP-MS strategy to determine potentially novel CRABP1 complexes in tissues/cells where CRABP1 is highly expressed, such as liver where atRA catabolism actively occurs [1, 44–46].

To determine whether exosome secretion can be affected by RA’s canonical activities, i.e. activities mediated by retinoic acid receptors (RARs), we compared the numbers of exosome in control and Crabp1 knockdown (siCrabp1)-p19 cultures treated with AGN193109, a pan-antagonist of RARs (Additional File 4: Supplementary Fig. 1). The data showed that blocking RA’s canonical activity in wild type P19 reduced the number of exosomes, indicating RA has a positive effect on exosome secretion. Interestingly, blocking RA’s canonical activity (AGN-treatment) in CRABP1-silenced group (siCrabp1 P19) reversed the reduction in exosome secretion, resulting in a slightly higher number of exosomes secreted from siCrabp1 p19 cells. Classical studies have proposed that CRABP1 could trap or channel RA to CYP450 enzymes for further metabolism [1, 44, 45], thereby reducing RA bioavailability. Therefore, depleting or reducing CRABP1 would probably increase RA bioavailability, and thus enhancing exosome secretion (as reflected in reversing the inhibitory effect of AGN treatment). However, given the complexity and redundant nature of RAR effects, further studies are needed to carefully examine these mechanisms.

Regulation of exosome release involves a complex interplay of various cellular signaling pathways, including calcium dynamics and protein kinase activity. Previous studies have shown that the formation of the CRABP1-CaMKII signalosome attenuates calmodulin-induced CaMKII activation, contributing to CRABP1’s function in modulating the health of MNs [10] and cardiomyocytes [6]. However, whether the specific impact of CRABP1 on exosome release involves calcium regulation remains to be experimentally tested. Moreover, the involvement of CRABP1-modulated kinase pathways in calcium regulation would suggest a broader network of signaling mechanisms that could involve CRABP1. The apparent trend of kinases appearing in CRABP1-interacting partners would suggest that kinases are probably one of important effector proteins within the branches of the CRABP1 interactome. For instance, certain kinase signaling pathways related to the regulation of exosome secretion also appeared in our IP-MS protein list (Table 1, Additional File 1: Supplemental Table 1), and we have validated that CRABP1 indeed formed complex with PKA (Fig. 5D) through its regulatory subunit. This is consistent with our previous findings that CRABP1 interacts with regulatory elements of kinases to modulate enzyme activity, such as the regulatory domain of CaMKII [47] and the Ras-binding domain (RBD) of Raf-1 [48].

We hypothesize that CRABP1 is important in exosome secretion to maintain the physiological homeostasis and cellular health. Exosome secretion provides a novel means of intercellular communication that facilitates the transport of biological cargo between cells. Therefore, exosome secretion itself is necessary for maintaining communication between cells required for health and physiological homeostasis [42]. Our studies of spinal cord motor neurons have shown that depleting CRABP1 indeed severely affected the health (such as the stress response) [49] and function (neuromuscular junction and synapse formation) of motor neurons [10]. Whether and how this new functional role of CRABP1 in regulating exosome secretion is directly or causally related to stress response and/or survival of motor neurons requires further studies. Interestingly, gene expression data of CKO tissues indicated that exosome biogenesis might also have been affected. The precise mechanisms and organelle dynamics, such as those between the trans-Golgi network, packaging and sorting of exosome cargos from extracellular and/or intracellular origins are currently under intense investigation in the field [50]. This current study has added an additional component/pathway that can participate in these complicated cellular processes.

This study underscores the ever-expanding functional roles of CRABP1, and supports the concept that CRABP1 forms various CRABP1-signalosomes to timely modulate various physiological processes to maintain optimal cellular health and function. Additionally, the ability to target specific CRABP1-signalosomes through CRABP1-selective (without activating RARs) compounds would provide an exciting avenue in designing novel therapeutics without retinoid toxicities. These results also shed new insights into the design and direction of future studies that can be tailored to address a particular process or pathway and that can be more physiologically and clinically significant in terms of CRABP1’s physiological function.

**Table 1:** Kinases and kinase-associated CRABP1-Interaction candidates

Gene	Protein Name	Associated Kinase(s)
PRKACA	cAMP-dependent protein kinase catalytic subunit alpha (PKA)	PKA [51]
PIK3R1	Phosphatidylinositol 3-kinase (PI3K) regulatory subunit alpha (p85)	PI3K [52]
PPP1CB	Serine/threonine-protein phosphatase (PP1)	MAPK [53, 54], PKA [55], GSK-3 [56], CDK [57–59], PI3K/AKT [60], and many others [61]
PIN1	Peptidyl-prolyl cis-trans isomerase NIMA-interacting 1 (PIN1)	MAPK [62, 63], CDK [62, 64], PKA [65], PKC [66], AKT [62], GSK-3 [62, 64]
GLRX3	Glutaredoxin-3/ PKC-interacting cousin of thioredoxin (PICOT)	PKC [67], JNK [67], ATR [68], Chk1/2 [68]
SFN	14-3-3 protein sigma/ Strafilin	MAPK [69], PKC [70], AKT [71], CDK [72], Chk1 [73]
RACK1	Receptor for activated C kinase 1 (RACK1)	PKC [74]
TWF2	Twinfilin-2	PKC [75]
EZR, RDX, MSN	Ezrin, Radixin and Moesin	PKC [76, 77], ROCK [78], LOK [79], NIK [80], PKA [81], and many others [43]
HNRNPA0	Heterogeneous nuclear ribonucleoprotein A0	MK2 [82]

**Kinase Abbreviations**: PKA (Protein kinase A); PI3K (Phosphoinositide 3-kinase); MAPK (Mitogen-activated protein kinase); GSK3 (Glycogen synthase kinase-3), CDK (Cyclin dependent kinase); AKT (also known as Protein Kinase B, PKB); PKC (Protein kinase C); JNK (c-Jun N-terminal kinases); ATR (Ataxia telangiectasia and Rad3-related); Chk1/2 (Checkpoint Kinase 1/2); ROCK (Rho-associated protein kinase); LOK (lymphocyte-oriented kinase); NIK (NF-kappa-B-inducing kinase); MAP kinase-activated protein kinase 2 (MAPK2)

## Methods

### Immunoprecipitation (IP)

HEK-293T cell (ATCC CRL-3216) line was maintained in complete DMEM medium (Gibco, #11965) containing 100 U/mL penicillin, 100 mg/mL streptomycin, and 10% heat-inactivated FBS. Flag-IP performed as described in [13]. Briefly, Control Flag-HA empty vector or Flag-HA-CRABP1 was transfected into HEK293T cell using the calcium phosphate method [83]. Depletion of endogenous hormones, including atRA, was performed by exchanging transfected HEK293T cells into dextran-charcoal-coated (DCC) containing median 24 h prior to harvesting. Cells were harvested using and the pull-down reaction performed in Co-IP buffer (50 mM Tris-HCl, pH 8.0, 150 mM NaCl, 10 mM MgCl2, 0.2% (v/v) NP-40, and 10% (v/v) glycerol) using M2-Flag agarose beads (Sigma, A2220). IP samples from Control Flag-HA and Flag-HA-CRABP1 lysates were the submitted to the University of Minnesota Center for Metabolomics and Proteomics (CMSP) Facility for sample preparation and mass spectrometry.

### IP-MS analysis pipeline

Raw files were analyzed using MaxQuant (v2.4.9) [18] as previously described searching against the UnitProt human reference proteome with canonical and isoform sequences [84, 85]. In brief, we imputed the data at the peptide level, then averaged intensity values for peptides that mapped to the same protein. In cases where the peptide mapped to multiple proteins or isoforms, MaxQuant algorithms identified the most likely protein candidate. We then performed variance-stabilized normalization (VSN) [86, 87].

A total of 436 protein were identified in the Control Flag-HA condition and 422 proteins were identified in the Flag-HA-CRABP1 condition. 377 of these proteins overlapped in both CF and Flag-CRABP1 conditions. 59 proteins were unique in to CF and 45 proteins were unique to the Flag-CRABP1 condition for a total of 481 proteins assessed for CRABP1 enrichment. Imputation followed by variance-stabilized normalization of intensity values allowed for direct quantitative comparisons to calculate CRABP1 enrichment values. Enrichment values for each identified protein was calculated by taking the numerical difference between the imputed, normalized intensity values in the Flag-HA-CRABP1 condition minus the intensity of Control Flag-HA condition (I _Flag−HA−CRABP1_ – I _Control Flag−HA_). An enrichment value > 0 indicated CRABP1 enrichment. Enrichment values < 0 indicate enrichment in the CF control condition, and values = 0 indicate no enrichment. Proteins with enrichment values ≤ 0 were excluded. A total of 240 candidates were identified. False positive screening was performed by querying CRABP1 enriched proteins in the Contaminant Repository for Affinity Purification (CRAPome) database [19]. A false positive was defined was as having a spectral count falling within the 4th quartile of all spectral counts observed in a single, relevant experiment across three independent experiments. Relevant experiments were defined by following criteria: (1) performed in a HEK293 background, (2) Flag or Flag-HA pull-down method, (3) and agarose bead support type. After false positive screening, a total of 86 proteins were identified as true CRABP1-candidate proteins. A complete list of the identified proteins pre-imputation and pre-normalization and the final list of protein candidates are available in Additional file 1: Supplementary Table 1.

The 86 candidate proteins were submitted to the Database for Annotation, Visualization and Integrated Discovery (DAVID) [20, 21] web server for GO Term Enrichment, Functional Annotation Clustering, KEGG pathway analysis [30–32] and Biocarta pathway analysis [33]. Default DAVID parameters were used. Significance cut-offs were set according to a Benjamini corrected *p*-value ≤ 0.05. The significance cut-off for functional clusters identified from Functional Clustering Annotation were set according to an enrichment score ≤ 1.3. A complete list of DAVID results is available in Additional file 2: Supplementary Table 2.

### Spinal cord collection

Mice were euthanized by CO2 asphyxiation. Immediately mice were processed for spinal cord isolation. The lumbar region of the spinal cord was flushed out with butterfly needle with PBS-filled 10 mL syringe.

### Quantitative RT-PCR

RNA was extracted by TRIzol Reagent (Ambion). RNA concentration was measured with the NanoDrop and cDNA was synthesized by High-Capacity cDNA Reverse Transcription Kit (Applied Biosystems™ #4368814). Quantitative RT-PCR was performed using the SYBR™ Green PCR Master Mix (ThermoFisher, # K0253). Real-time RT-PCR was conducted on QuantStudio™ 3 Real-Time PCR System (Applied Biosystems™). Primers listed in Additional File 3: Supplemental Table 3.

### Nanoparticle tracking analysis, NTA

Exosomes were isolated using Total Exosome Isolation Reagent (Invitrogen, # 4478360) according to the manufacturer’s instructions, and the pellet was resuspended in PBS. NTA was performed using a Nanoparticle Tracking Analyzer (Model: NanoSight LM-10) provided by University of Minnesota Nano Center. The particle size distribution of nanoparticles with diameters of 10–200 nm.

### Cerebrospinal fluid, CSF isolation

The CSF collection was performed as described previously protocol [7]. Briefly, after anesthetizing the mice with CO2, the skin and musculature was displaced until the meninges on top of the cisterna magna were exposed. The CSF was immediately collected from the cisterna magna using the custom-made calibrated micropipette (Drummond Scientific, #2-000-050). Clear CSF were collected from 3 mice and pooled together. Blood-contaminated samples were not used. CSF samples were stored in − 20 °C until use.

### Isolation of exosomes for flow cytometry

Exosomes were isolated from PS Capture™ Exosome Flow Cytometry Kit (FUJIFILM Wako Chemicals, #297-79701) according to the manufacturer’s instructions. In brief, CSF, serum or cells supernatant were incubated with exosome binding enhancer and exosome capture beads at room temperature for 1 h. Exosomes were washed and resuspend in wash buffer for immunostaining. Exosome surface marker CD9 (Biolegend, clone: MZ3) and isotype control (Biolegend, #400511) were labeled on ice for 30 min, then washed and resuspend in FACS buffer and analyzed by BD™ LSR II flow cytometry and analyzed by the FlowJo^®^ software. Absolute numbers of cells were determined by comparing the Precision Count Beads (Biolegend, #424902) count and cell count.

### Stable cell line generation

HEK-293T (ATCC CRL-3216) cell line was cultured in complete DMEM medium (Gibco, #11965) containing 100 U/mL penicillin, 100 mg/mL streptomycin, and 10% heat-inactivated FBS. P19 cell lines (ATCC, #CRL1825) was cultured in complete MEMα medium (Gibco, #12571-063) containing 100 U/mL penicillin, 100 mg/mL streptomycin, and 10% serum containing 7.5% bovine calf serum, ion fortified (ATCC, #30-2030) and 2.5% FBS (R & D Systems, #S11195). For exosome collection, cell was cultured in medium supplemented with 10% exosome depleted FBS (Gibco, A2720801). Cells were regularly tested for mycoplasma contamination.

pLKO.1-shCrabp1 (TRCN0000011959 and TRCN0000011960) and pLKO.1 vector plasmids were purchased from UMGC RNAi (University of Minnesota Genomics Center, RNA Interference). For lentivirus production, 2 × 10^6^ HEK-293T cells were seeded in complete DMEM medium without antibiotics dish in 10 cm dish overnight. 9.6 µg target plasmid, 7.2 µg psPAX2 packaging plasmid, 2.4 µg pMD2.G envelope plasmid was co-transfected into cells with Lipofectamine 2000 transfection reagent (Invitrogen) following the manufacturer’s protocol. Changed media to fresh complete DMEM medium containing 1% BSA after 6 h. Infectious lentiviruses were harvested at 24 h and 48 h post-transfection and filtered through 0.45 µM pore cellulose acetate filters. Subsequently, concentrating lentiviral stocks by using Lenti-X Concentrator (Clontech Labs, #631232) according to the manufacturer’s protocol.

For transduction, 2 × 10^5^ P19 cells were seeded in complete DMEM medium in 6 well plate overnight. For P19-siCrabp1, lentivirus derived from TRCN0000011959 and TRCN0000011960 clones, along with 8 µg/ml polybrene (Millipore TR-1003-G), were introduced to the cells. In contrast, for P19-control, lentivirus from the pLKO.1 vector with 8 µg/ml polybrene were added to the cells. The cells were then subjected to centrifugation at 800 g, 37 °C for 60 min. Lentivirus was removed and changed fresh medium after 24 h, and started the puromycin selection at 48 h post transfection. Cells were selected and maintained in the same medium as described above with the addition of 1.5 µg/ml puromycin. For single cell clone isolation, 10 cells were seeded in a 10-cm dish. Following one week, colonies reached a size visible to the naked eye. These colonies were then picked using 20 µl of trypsin and transferred into 96-well plate. Stable P19 clones were subsequently collected and Crabp1 expression was examined by qPCR.

### Statistical analysis

No animals were excluded from the analyses. Two-tailed Student’s t-test was used when appropriate for comparison among the groups. Data were normally distributed, and variance was similar among groups that were being statistically compared. Data were presented as means ± SD. The comparison was considered statistically significant when *p* values ≤ 0.05 (* *p* < 0.05; * * *p* < 0.01; * * * *p* < 0.001). Excel and Prism 6.0 (GraphPad) was used for plotting data and statistical analysis.

### His-CRABP1 pull-down assay and western blot

His-CRABP1 was purified as described in [15], and served as the bait protein. His-CRABP1 (5 mM) bait protein was immobilized to nickel-nitrilotriacetic acid resin (Ni-NTA, Qiagen) in a total volume of 500 ul of reaction buffer (50 mM Tris pH 8.0, 150 mM NaCl, 0.2% (v/v) NP-40, 20% glycerol, and 50 mM imidazole) for 1 h at 4 °C with agitation.

P19 lysate provided endogenous source for ARPC3 and PKA RI-alpha prey proteins. P19 cells were subjected to whole-cell lysate protein extraction, by resuspending pelleted cells in lysis buffer (50 mM Tris pH 8.0, 150 mM NaCl, 0.2% (v/v) NP-40, 20% glycerol, and 1X protease–phosphatase inhibitor solution (Cell Signaling Cat # 58725)). The cell lysate was then centrifuged at high speed (16,000 x g, 15 min, 4 °C) to remove debris. Cell lysate protein extract was quantified using Bradford assay with Bradford reagent (Bio-Rad Cat # 5,000,001) on a Bio-Rad Smart Spec spectrometer. P19 lysate was pre-cleared by incubation with Ni-aresin for 1 h at 4 °C with agitation.

For each pull-down reaction, immobilized His-CRABP1 was incubated with 1000 ug of pre-cleared P19 lysate in reaction buffer (50 mM Tris pH 8.0, 150 mM NaCl, 0.2% (v/v) NP-40, 20% glycerol, 50 mM imidazole, and 1X protease–phosphatase inhibitor solution) overnight with agitation at 4 °C. Pull-down reactions were then washed for 30 s with agitation, five times with wash buffer (50 mM Tris pH 8.0, 150 mM NaCl, 0.2% (v/v) NP-40, 20% glycerol, and 20 mM imidazole). The reaction was terminated by removing the wash buffer and resuspending the reaction Ni-NTA beads in SDS lysis buffer (9 parts: 128 mM Tris base, 10% (v/v) glycerol, 4% (w/v) SDS, 0.1% (w/v) bromophenol blue, pH to 6.8 and 1 part: beta-mercaptoethanol).

Western blot was performed as described in [88], with the following modifications: reactions were separated using a 4–20% SDS PAGE polyacrylamide gel (Bio-Rad #4,561,094) and transferred onto a 0.25 μm PVDF membrane (Thermo Fisher Scientific #88520). The following primary antibodies and dilutions were used: Anti-APRC3 (Fisher Scientific # 50-157-0369, 1:1000), Anti-PKA RI-alpha (Cell Signaling #3927, 1:1000), Anti-His Probe (Santa Cruz #SC-8036, 1:1000). The following secondary anti-bodies and dilutions were used: anti-Rabbit-IgG-HRP (Jackson ImmunoResearch #11-035-144, 1:2000) and anti-Mouse-IgG-HRP (GeneTex #GTX26789, 1:5000).

It should be noted that some background binding of prey proteins was observed in the negative control, a previously known phenomena [89]. These non-specific interactions may be due to interactions with the nickel metal ion or the agarose resin support itself. The addition of relatively high concentrations of imidazole (50 mM) as a blocking agent and extensive washing of the reaction beads was performed in order to reduce this nonspecific, background binding as much as possible.

### Electronic supplementary material

Below is the link to the electronic supplementary material.

Supplementary Material 1. Name: Additional File 1_Supplementary Table 1. File format: .xlsx. Title: Supplementary Table 1. Complete Protein List and Pre-imputed, Pre-normalized Peptide and Protein Intensities. Description: Complete Protein list from MaxQuant Output and pre-imputed, pre-normalized peptide and protein intensities.

Supplementary Material 2. Name: Additional File 2_Supplementary Table 2. File format: .xlsx. Title: Supplementary Table 2. Complete GO enrichment, Functional Annotation Clustering, KEGG and Biocarta Pathway Analysis Results. Description: Raw output from DAVID web server for Complete GO enrichment, Functional Annotation Clustering, KEGG and Biocarta Pathway Analysis.

Supplementary Material 3. Name: Additional File 3_Supplementary Table 3. File format: .pdf. Title: Supplementary Table 3. List of primers for quantitative RT-PCR. Description: Primer sequences for gene markers used in gene expression qPCR experiments.

Supplementary Material 4. Name: Additional File 4_Supplementary Fig. 1. File format: .pdf. Title: Supplementary Fig. 1. Flow cytometry analyses of exosomes collected from supernatant of P19 cells. Description: Cells were pretreated with DMSO or 1 µM AGN193109 for 1 h. Following pre-treatment, the medium was replaced with exosome-depleted medium, and supernatants were collected after 24 h. Error bars show means ± SEM. One-way ANOVA, *p < 0.05, **p < 0.01. MFI = Mean fluorescence intensity.

## Data Availability

Data is provided within the manuscript or supplementary information files.

## Abbreviations

ACTR2: Actin Related Protein 2
ACTR3: Actin Related Protein 3
AKT: Protein Kinase B
ALIX: Programmed cell death 6-interacting protein
AMP: adenosine monophosphate
ARP2: Actin Related Protein 2
ARPC3: Actin Related Protein 2/3 Complex Subunit 3
ARPC4: Actin Related Protein 2/3 Complex Subunit 4
BSA: Bovine Serum Albumin
CD63: CD63 Antigen
CD9: CD9 Antigen
CDC42: Cell Division Cycle 42
CDK: Cyclin Dependent Kinase
CF: Control Flag-HA
CHMP4B: Charged Multivesicular Body Protein 4B
CRABP1: Cellular Retinoic Acid Binding Protein 1
CKO: CRABP1 Knockout
CMSP: Center for Mass Spectrometry and Proteomics
CSF: Cerebral Spinal Fluid
DAVID: Database for Annotation, Visualization and Integrated Discovery
ERM: Ezrin-Radixin-Moesin
ESC: Embryonic Stem Cell
ESCRT: endosomal sorting complexes required for transport
EZR: Ezrin
FDR: False Detection Rate
GLRX3: Glutaredoxin 3
GO: Gene Ontology
GSK3: Glycogen synthase kinase 3
HNRNPA0: Heterogeneous nuclear ribonucleoprotein A0
JNK: c-Jun N-terminal kinases
KEGG: kyoto encyclopedia of genes and genomes
LOK: lymphocyte-oriented kinase
MAPK: Mitogen Activated Kinase
MAPK2: MAP kinase-activated protein kinase 2
MFI: mean fluorescence intensity
MK2: MAPKAP Kinase-2
MN: motor neurons
MS: Mass Spectrometry
MSN: Moesin
MVB: multivesicular bodies
NMJ: neuromuscular junction
NSC: neural stem cell
Ni-NTA: nickel-nitrilotriacetic acid
NTA: Nanoparticle Tracking Analysis
PI3K: Phosphatidylinositol 3-kinase
PICOT: Glutaredoxin-3/ PKC-interacting cousin of thioredoxin
PIK3R1: Phosphatidylinositol 3-kinase (PI3K) regulatory subunit alpha (p85)
PIN1: Peptidyl-prolyl cis-trans isomerase NIMA-interacting 1
PKA: Protein kinase A
PKA-RI: Protein Kinase A Regulatory Subunit
PKB: Protein kinase B
PKC: Protein kinase C
PP1: Protein phosphatase 1
PPP1CB: Protein Phosphatase 1 Catalytic Subunit Beta
PRKACA: Protein Kinase CAMP-Activated Catalytic Subunit Alpha
RA: retinoic acid
RAB11: RAB11, Member RAS Oncogene Family
RAB3A: RAB3A, Member RAS Oncogene Family
RAB5: RAB5A, Member RAS Oncogene Family
RAB7: RAB7A, Member RAS Oncogene Family
RACK1: Receptor for activated C kinase 1
RAR: RA receptors
RDX: Radixin
RIP140: Receptor-interacting protein 140
ROCK: Rho-associated protein kinase
RPL19: Ribosomal Protein L19
SFN: 14-3-3 protein sigma/ Strafilin
SNARE: Soluble N-ethylmaleimide-sensitive factor attachment proteins receptors
TNFR1: TNF Receptor 1
TSG101: Tumor Susceptibility 101
TWF2: Twinfilin-2
VSN: variance-stabilized normalization
WNT10: Wnt Family Member 10
