# Supplementary material for: CRABP1-complexes in exosome secretion
Source: Cell Commun Signal. 2024 Jul 29;22:381. doi: 10.1186/s12964-024-01749-w (PMC11285139; doi:10.1186/s12964-024-01749-w)
Supplement: Supplementary file 3 — Supplementary Material 3. Name: Additional File 3_Supplementary Table 3. File format: .pdf. Title: Supplementary Table 3. List of primers for quantitative RT-PCR. Description: Primer sequences for gene markers used in gene expression qPCR experiments. [file 12964_2024_1749_MOESM3_ESM.pdf]

Table S3. List of primers for quantitative RT-PCR

Gene Name	Primer Sequence (Forward)	Primer Sequence (Reverse)
CD63	GGAATCCACTATCCATACCCAGG	TCTTCACCAGACAGCAGGAGA
CD9	CTGTGGCATAGCTGGTCCTTTG	AGACCTCACTGATGGCTTCAGG
Cell Division Cycle 42 (CDC42)	GATTGGTGGAGAGCCATACAC	TGAGGATGGAGAGACCACTG
Cellular retinoic acid binding protein 1 (Crabp1)	5’ CGGAGATCAACTTCAAGGTCGG 3’	5’ CCCTCAAGAAGTGTCTGTGTGC 3’
Charged Multivesicular Body Protein 4B (CHMP4B)	CAGGACATTGCTGACCAGCA	AGTTCCTCCAACTCTGCCATG
Fibroblast Growth Factor 2 (FGF2)	AGCGGCTCTACTGCAAGAAC	CCTTGATAGACACAACTCCTCTC
Hepatocyte Growth Factor-Regulated Tyrosine Kinase Substrate (HRS)	TCTCAGCCCATAACTCCCTGC	CGGTTGACAAAAGTGCTCACG
Programmed Cell Death 6 Interacting Protein,PDCD6IP (ALIX)	GGACTCCATCCAATGACCTGT	CGGCTTACACAGAAGTGCGA
RAB11A, Member RAS Oncogene Family (RAB11a)	CAAGAAGCATCCAGGTTGATGG	AAGGCACCTACTGCTCCAC
RAB11B, Member RAS Oncogene Family (RAB11b)	TGAGACCTCAGCCTTGGATTC	GGTCAGCGATTTGCTTCTGTG
RAB27A, Member RAS Oncogene Family (RAB27a)	AGCAAAGTTTCCTCAATGTCCG	CTTTCACTGCCCTCTGGTCT
RAB27B, Member RAS Oncogene Family (RAB27b)	CCTCACCAGTCAACAGAGCT	CCGTTCATTGACTTCCCTTTGG
RAB31, Member RAS Oncogene Family (RAB31)	CCAAAACCGTGCCTTGTGGA	CATAGACGATGACGGCTGCA
RAB35, Member RAS Oncogene Family (RAB35)	GGTGGTAGAGACAGAAGATGCC	TCTTTGCTCGCAGAACCAGC
RAB3A, Member RAS Oncogene Family (RAB3a)	GCTACGCAGATGACTCCTTCA	CGGTAATAGGCTGTGGTGATG
RAB5A, Member RAS Oncogene Family (RAB5)	AGCACAGTCCTATGCAGATGAC	GCTGAGTTTGCACCAGGATTC
RAB7A, Member RAS Oncogene Family (RAB7)	GCGGACTTTCTGACCAAGGA	ATCTGCACCTCTGTAGAAGGC
RAS Like Proto-Oncogene A (RalA)	ACAGAGCTGACCAGTGGAAC	GCTGTCTTCCATCTTTCTGGC
Ribosomal Protein L19 (RPL19)	5’ TCATCCGCAAGCCTGTGACT 3’	5’ CTTCTCAGGCATCCGAGCAT 3’
Tumor Susceptibility 101 (TSG101)	GGTTATCCTGGCTGTCCTTACC	GCACGGATAGTGTCCTCACTGA
Vacuolar Protein Sorting 4 Homolog B (VPS4B)	GAGCGACCAAATGTGAAGTGGAG	TCCTCTCCAAGGTGTCCGC
Vesicle Associated Membrane Protein 3 (VAMP3)	AGACCAGAAGCTCTCGGAGC	ACCAGGACACTGATCCCTATCG
Vesicle Associated Membrane Protein 3 (VAMP5)	AGCTGCTCTTTGGTTAAAAATGC	GTGTAATGCCTATGTCTGGTGT
Vesicle Associated Membrane Protein 7 (VAMP7)	CCAGACAACTTACGGTTCAAGAG	CCACTTGTGCTTGAGTCTCC
VPS37B Subunit Of ESCRT-I (VPS37B)	CCAACTGGATGCTCAGAAAGC	AGAGTCTCCAAGGAAGCGTTG
YKT6 V-SNARE Homolog (YKT6)	AAGGCAGCAGAGCTTCTGT	GCTGTCAGCAATGACCACAC
