# Supplementary material for: CRABP1-complexes in exosome secretion
Source: Cell Commun Signal. 2024 Jul 29;22:381. doi: 10.1186/s12964-024-01749-w (PMC11285139; doi:10.1186/s12964-024-01749-w)
Supplement: Supplementary file 4 — Supplementary Material 4. Name: Additional File 4_Supplementary Fig. 1. File format: .pdf. Title: Supplementary Fig. 1. Flow cytometry analyses of exosomes collected from supernatant of P19 cells. Description: Cells were pretreated with DMSO or 1 µM AGN193109 for 1 h. Following pre-treatment, the medium was replaced with exosome-depleted medium, and supernatants were collected after 24 h. Error bars show means ± SEM. One-way ANOVA, *p < 0.05, **p < 0.01. MFI = Mean fluorescence intensity. [file 12964_2024_1749_MOESM4_ESM.pdf]

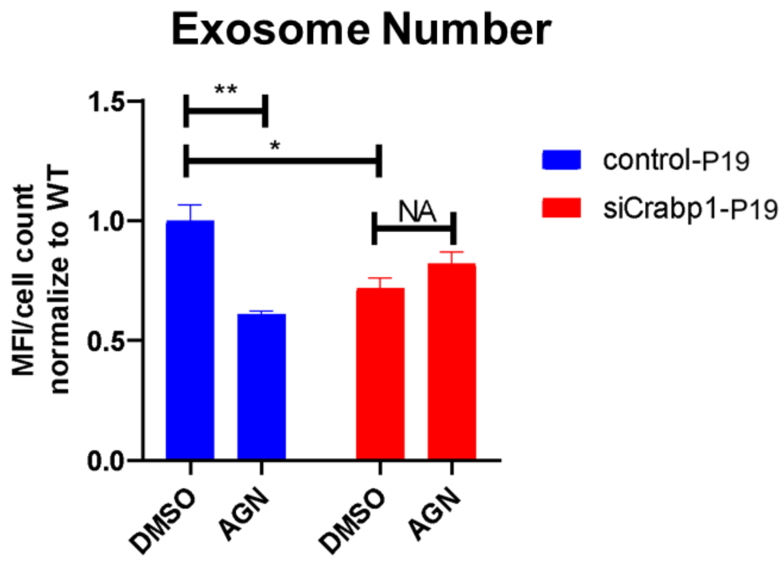

Supplementary Figure 1. Flow cytometry analyses of exosomes collected from supernatants of AGN-treated P19 cells. Cells were pre-treated with DMSO or 1 µM AGN193109 for 1 hour. Following pre-treatment, the media was replaced with exosome-depleted medium, and supernatants were collected after 24 hours. Error bars show mean ± SEM. One-way ANOVA, *$p<0.05$, **$p<0.01$. MFI = Mean fluorescence intensity.
